# Supplementary material for: PreBötzinger complex neurons drive respiratory modulation of blood pressure and heart rate
Source: eLife. 2020 Jun 15;9:e57288. doi: 10.7554/eLife.57288 (PMC7326498; doi:10.7554/eLife.57288)
Supplement: Transparent reporting form [file elife-57288-transrepform.docx]

***eLife’s* transparent reporting form**

We encourage authors to provide detailed information *within their submission* to facilitate the interpretation and replication of experiments. Authors can upload supporting documentation to indicate the use of appropriate reporting guidelines for health-related research (see [EQUATOR Network](http://www.equator-network.org/%20)), life science research (see the [BioSharing Information Resource](https://biosharing.org/)), or the [ARRIVE guidelines](http://www.plosbiology.org/article/info:doi/10.1371/journal.pbio.1000412) for reporting work involving animal research. Where applicable, authors should refer to any relevant reporting standards documents in this form.

If you have any questions, please consult our Journal Policies and/or contact us: [editorial@elifesciences.org](mailto:editorial@elifesciences.org).

**Sample-size estimation**

- You should state whether an appropriate sample size was computed when the study was being designed
- You should state the statistical method of sample size computation and any required assumptions
- If no explicit power analysis was used, you should describe how you decided what sample (replicate) size (number) to use

Please outline where this information can be found within the submission (e.g., sections or figure legends), or explain why this information doesn’t apply to your submission:

No statistical methods were used to pre-determine sample sizes. Our sample sizes are based on years of experience using experimental preparations and protocols similar to those used in this study, and are similar to those used by others in the field.

**Replicates**

- You should report how often each experiment was performed
- You should include a definition of biological versus technical replication
- The data obtained should be provided and sufficient information should be provided to indicate the number of independent biological and/or technical replicates
- If you encountered any outliers, you should describe how these were handled
- Criteria for exclusion/inclusion of data should be clearly stated
- High-throughput sequence data should be uploaded before submission, with a private link for reviewers provided (these are available from both GEO and ArrayExpress)

Please outline where this information can be found within the submission (e.g., sections or figure legends), or explain why this information doesn’t apply to your submission:

Sample numbers are provided in the methods section, in each figure legend, and precisely described in the data source and statistics associated files. Technical replication is commented at the end of the “Optogenetic stimulation” paragraph in the methods section. For Working Heart-Brainstem Preparation experiments, only preparations that retained physiological patterns of respiratory and sympathetic nerve activities throughout the recording period were kept for analysis (6 GtACR2 and 1 ChR2 preparations were excluded). These patterns are pre-determined indicators of validity and viability of the experimental preparation. Within preparations, the order of the various photostimulation protocols was randomised, and if the recording of a single nerve was degraded for a specific part of the protocol, then this specific component of the protocol was excluded. For instance, electrical noise issues affected vagus nerve recordings in several preparations, in particular during some 5Hz and 50Hz GtACR2-mediated photoinhibitions, and the records of activity in this nerve was excluded from the analysis.

**Statistical reporting**

- Statistical analysis methods should be described and justified
- Raw data should be presented in figures whenever informative to do so (typically when N per group is less than 10)
- For each experiment, you should identify the statistical tests used, exact values of N, definitions of center, methods of multiple test correction, and dispersion and precision measures (e.g., mean, median, SD, SEM, confidence intervals; and, for the major substantive results, a measure of effect size (e.g., Pearson's r, Cohen's d)
- Report exact p-values wherever possible alongside the summary statistics and 95% confidence intervals. These should be reported for all key questions and not only when the p-value is less than 0.05.

Please outline where this information can be found within the submission (e.g., sections or figure legends), or explain why this information doesn’t apply to your submission:

Statistical methods are described in the methods section, as well as in each figure legend. All detailed statistical analysis are provided in the associated data source and statistics files with each figure. All raw data are presented, either in the primary figures, or in the associated supplementary figures.

(For large datasets, or papers with a very large number of statistical tests, you may upload a single table file with tests, Ns, etc., with reference to sections in the manuscript.)

**Group allocation**

- Indicate how samples were allocated into experimental groups (in the case of clinical studies, please specify allocation to treatment method); if randomization was used, please also state if restricted randomization was applied
- Indicate if masking was used during group allocation, data collection and/or data analysis

Please outline where this information can be found within the submission (e.g., sections or figure legends), or explain why this information doesn’t apply to your submission:

Our experimental subjects, outbred Sprague-Dawley rats, were obtained from a genetically homogeneous colony. Also, we mostly used paired experiments, so we didn’t have to allocate animals to different groups as they were their own controls. In this context, randomization is not relevant to this study.

Since we performed paired experiments, blinding of the experimenter was not relevant to this study. The analyser was blinded to the expression profile of the opsin used, assessed a posteriori to determine inclusion/exclusion of the data.

**Additional data files (“source data”)**

- We encourage you to upload relevant additional data files, such as numerical data that are represented as a graph in a figure, or as a summary table
- Where provided, these should be in the most useful format, and they can be uploaded as “Source data” files linked to a main figure or table
- Include model definition files including the full list of parameters used
- Include code used for data analysis (e.g., R, MatLab)
- Avoid stating that data files are “available upon request”

Please indicate the figures or tables for which source data files have been provided:

Source data and statistics files have been provided for all figures that include numerical data, i.e. figures 2, 3, 4, 5, 6, 7 and 8.
